# When the Heart Is the Target: Case of Primary Pericardial Diffuse Large B‐Cell Lymphoma Presenting With Tamponade

**DOI:** 10.1155/crom/9885772

**Published:** 2026-01-16

**Authors:** Ameed Bawwab, Lana Khatib, Moath Ahmad Salman Albliwi, Linlin Yang, Pei Jun Zhao

**Affiliations:** ^1^ Department of Internal Medicine, Cleveland Clinic Mercy Hospital, Canton, Ohio, USA, clevelandclinic.org; ^2^ Department of Internal Medicine, Cleveland Clinic Foundation, Cleveland, Ohio, USA, clevelandclinic.org; ^3^ Department of Pathology, Cleveland Clinic Mercy Hospital, Canton, Ohio, USA, clevelandclinic.org; ^4^ Cleveland Clinic Lerner School of Medicine, Case Western Reserve University, Cleveland, Ohio; Western University, London, Canada, case.edu

**Keywords:** diffuse large B-cell lymphoma, non-Hodgkin lymphoma, pericardial effusion

## Abstract

Diffuse large B‐cell lymphoma (DLBCL) is an aggressive malignancy and the most common subtype of non‐Hodgkin lymphoma, typically presenting with lymphadenopathy. Pericardial involvement is extremely rare. We report the case of a patient who presented with cough and shortness of breath and was admitted for acute hypoxic respiratory failure. During hospitalization, the patient developed atrial fibrillation with rapid ventricular response (RVR). Transthoracic echocardiography revealed a pericardial effusion, which increased in size on repeat imaging 1 week later. The patient subsequently underwent pericardiocentesis, and cytology of the pericardial fluid confirmed DLBCL, with the pericardium identified as the primary site of involvement. The patient was treated with mini‐R‐CHOP chemotherapy, resulting in marked clinical improvement.

## 1. Introduction

Diffuse large B‐cell lymphoma (DLBCL) is a heterogeneous and aggressive malignancy, representing the most common subtype of non‐Hodgkin lymphoma [1]. It typically presents with single or multiple lymphadenopathies, although nearly 50% of cases exhibit extranodal involvement—most commonly in the skin and gastrointestinal tract. However, cardiac involvement, particularly isolated pericardial disease, is exceedingly rare [2, 3]. Pericardial effusion can arise from various etiologies across all age groups, most commonly viral pericarditis. Malignancy accounts for approximately 25% of pericardial effusions, predominantly due to solid tumors (lung, gastrointestinal, and renal) [4]. Hematologic malignancies such as lymphoma are far less frequently implicated.

We report a case of an 81‐year‐old male who presented with cough, shortness of breath, and fatigue and was found to have a large pericardial effusion. Further workup revealed a diagnosis of DLBCL, and the patient was subsequently started on mini‐R‐CHOP chemotherapy.

## 2. Case Presentation

An 81‐year‐old male with a past medical history of chronic kidney disease Stage 3, hypertension, hyperlipidemia, and benign prostatic hyperplasia presented with a 2‐week history of fatigue, nonproductive cough, shortness of breath, myalgias, and bilateral lower extremity edema. In the emergency department, his vital signs were as follows: a blood pressure of 130/85 mmHg, a heart rate of 80 bpm, a respiratory rate of 18 breaths per minute, and an oxygen saturation of 85% on room air.

Initial evaluation revealed bibasilar airspace opacities on chest x‐ray. Influenza B PCR was positive, and the patient was admitted with a diagnosis of acute hypoxic respiratory failure secondary to viral pneumonia. On hospital Day 3, he developed new‐onset atrial fibrillation with rapid ventricular response. A transthoracic echocardiogram (TTE) performed on hospital Day 4 revealed a moderate, circumferential 2 cm pericardial effusion with preserved ejection fraction (EF 59%). The effusion was initially attributed to viral pericarditis. The patient was treated with oseltamivir, metoprolol for rate control, and anticoagulation. He improved clinically and was discharged in stable condition, with plans for outpatient cardiology follow‐up and repeat echocardiography in 1 week.

**Figure 1:**
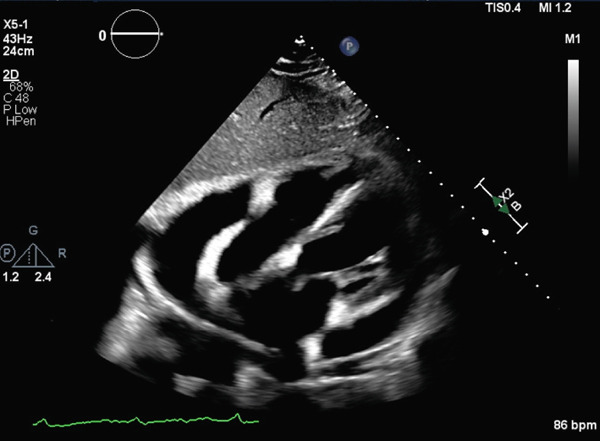
Echocardiographic image showing a large circumferential pericardial effusion with early tamponade physiology (right atrial diastolic collapse).

One week later, a repeat outpatient echocardiogram revealed a large pericardial effusion with early signs of tamponade (Figure 1).

**Figure 2:**
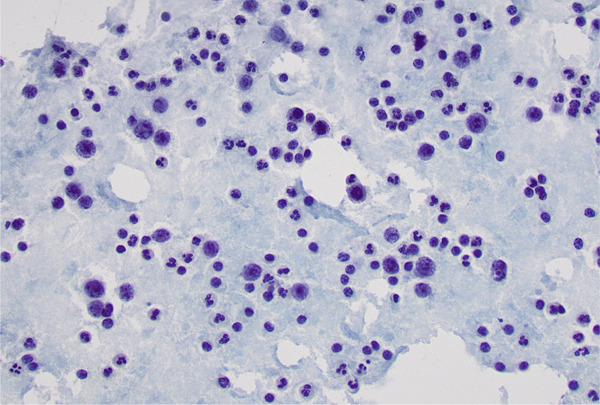
Cytopathology demonstrates large atypical lymphoid cells with vesicular chromatin, prominent nucleoli, and brisk mitotic activity—morphologically consistent with DLBCL.

Urgent pericardiocentesis yielded 930 cc of amber‐colored fluid. The fluid was sent for cytological and biochemical analysis (Table 1). Cytology revealed malignant cells. Pathology slides were reviewed independently by three pathologists, all of whom concurred with a diagnosis of DLBCL. The specimen contains abundant large lymphoid cells with vesicular chromatin, multiple variably prominent nucleoli, brisk mitosis, and apoptosis. Immunohistochemical stains performed on the cellblock show that the large tumor cells are strongly positive for CD20 and Bcl‐6, focally positive for MUM‐1, and negative for AE1/3, calretinin, CD30, and CD138. The stain of CD10 is equivocal with strong background staining of numerous granulocytes (Figure 2). Stains of CD3 and CD5 highlight abundant nonneoplastic small T‐lymphocytes. The Ki‐67 proliferation index was up to 80%. The overall findings are compatible with a high‐grade large B‐cell lymphoma.

**Table 1:** Biochemical analysis of the pericardial fluid showed.

**Test**	**Result**	**Normal range**
Color	Amber	Yellow
RBC	13,000/*μ*L (↑)	< 2000/*μ*L
Total nucleated cells	7074/*μ*L (↑)	< 1000/*μ*L
Neutrophils	61% (↑)	0%–1%
Lymphocytes	30%	18%–36%
Cytology	Malignant (DLBCL)	Negative
LDH	521	—
Glucose	86	—
Protein	5.7	—

Positron emission tomography (PET/CT) revealed mildly increased metabolic activity in the right paratracheal/precarinal lymph node and mildly metabolically active pericardial thickening, with a residual small‐volume pericardial effusion. Additional workup, including testing for Epstein–Barr virus (EBV), Human Herpesvirus 8 (HHV‐8), and a bone marrow biopsy, was unremarkable.

Based on cytology, imaging, and clinical findings, the patient was diagnosed with primary pericardial DLBCL. He was initiated on mini‐R‐CHOP, a dose‐reduced chemotherapy regimen appropriate for elderly or frail patients. Following three cycles, a repeat PET scan demonstrated resolution of metabolic activity in the previously affected lymph nodes and pericardial region, indicating an excellent early response to therapy. Repeat echocardiogram did not show reaccumulation of pericardial effusion.

Written informed consent was obtained from the patient for publication of this case report and accompanying clinical data and images.

According to the Cleveland Clinic IRB policy, single‐patient case reports are exempt from IRB review and do not require formal approval.

## 3. Discussion

Our case represents an uncommon presentation of DLBCL with primary pericardial involvement. Cardiac involvement in DLBCL often presents with nonspecific symptoms, including dyspnea, peripheral edema, pericardial effusion, and even heart failure, which can make early diagnosis challenging [5]. Such involvement is typically secondary, while primary cardiac lymphoma is exceedingly rare [3]. This rarity highlights the significance of our case and supports its contribution to the existing literature awareness.

Primary cardiac lymphoma exhibits rapid growth increasing mortality and risk of death if not treated promptly. In our patient, serial echocardiograms revealed progressive worsening of pericardial effusion over a short period. This prompted pericardiocentesis, and cytologic analysis of the pericardial fluid confirmed the diagnosis of DLBCL leading to early diagnosis and prompt treatment.

This case emphasizes the importance of considering malignancy in the differential diagnosis of unexplained, worsening, or large pericardial effusions, particularly when there is no obvious infectious or autoimmune cause as making.

The diagnosis of DLBCL is typically established through cytological and histopathological evaluation, with PET imaging playing a critical role in assessing the extent and distribution of disease. In our case, cytological analysis confirmed DLBCL, while the PET scan showed only mild metabolic activity, and the bone marrow biopsy was unremarkable, supporting the pericardium as the primary site of disease. The patient was started on mini‐R‐CHOP, a dose‐reduced regimen commonly used in elderly or frail patients with DLBCL [6].

## 4. Conclusion

Our case underscores the diagnostic challenges associated with pericardial involvement in DLBCL, especially due to its rarity and the nonspecific nature of symptoms such as fatigue and dyspnea. It emphasizes the critical role of early pericardial fluid analysis in establishing a prompt diagnosis. Given the aggressive nature of DLBCL, timely diagnosis and initiation of appropriate chemotherapy are essential to improving outcomes and preventing serious complications such as cardiac tamponade or disease progression.

## Ethics Statement

Institutional review board (IRB) approval was not required for this case report because it describes a single patient without identifiable information, in accordance with institutional policy and international case report guidelines.

## Conflicts of Interest

The authors declare no conflicts of interest.

## Funding

No funding was received for this manuscript.

## Data Availability

Data sharing is not applicable to this article as no datasets were generated or analyzed during the current study.
